# Consistency of electrical source imaging in presurgical evaluation of epilepsy across different vigilance states

**DOI:** 10.1002/acn3.51959

**Published:** 2024-01-12

**Authors:** Tamir Avigdor, Chifaou Abdallah, Jawata Afnan, Zhengchen Cai, Saba Rammal, Christophe Grova, Birgit Frauscher

**Affiliations:** ^1^ Analytical Neurophysiology Lab Montreal Neurological Institute and Hospital, McGill University Montreal Quebec Canada; ^2^ Multimodal Functional Imaging Lab, Biomedical Engineering Department McGill University Montreal Canada; ^3^ Montreal Neurological Institute and Hospital, McGill University Montreal Quebec Canada; ^4^ Multimodal Functional Imaging Lab, PERFORM Centre, Department of Physics Concordia University Montreal Quebec Canada; ^5^ Department of Neurology Duke University Medical Center Durham North Carolina USA; ^6^ Department of Biomedical Engineering Duke Pratt School of Engineering Durham North Carolina USA

## Abstract

**Objective:**

The use of electrical source imaging (ESI) in assessing the source of interictal epileptic discharges (IEDs) is gaining increasing popularity in presurgical work‐up of patients with drug‐resistant focal epilepsy. While vigilance affects the ability to locate IEDs and identify the epileptogenic zone, we know little about its impact on ESI.

**Methods:**

We studied overnight high‐density electroencephalography recordings in focal drug‐resistant epilepsy. IEDs were marked visually in each vigilance state, and examined in the sensor and source space. ESIs were calculated and compared between all vigilance states and the clinical ground truth. Two conditions were considered within each vigilance state, an unequalized and an equalized number of IEDs.

**Results:**

The number, amplitude, and duration of IEDs were affected by the vigilance state, with N3 sleep presenting the highest number, amplitude, and duration for both conditions (*P* < 0.001), while signal‐to‐noise ratio only differed in the unequalized condition (*P* < 0.001). The vigilance state did not affect channel involvement (*P* > 0.05). ESI maps showed no differences in distance, quality, extent, or maxima distances compared to the clinical ground truth for both conditions (*P* > 0.05). Only when an absolute reference (wakefulness) was used, the channel involvement (*P* < 0.05) and ESI source extent (*P* < 0.01) were impacted during rapid‐eye‐movement (REM) sleep. Clustering of amplitude‐sensitive and ‐insensitive ESI maps pointed to amplitude rather than the spatial profile as the driver (*P* < 0.05).

**Interpretation:**

IED ESI results are stable across vigilance states, including REM sleep, if controlled for amplitude and IED number. ESI is thus stable and invariant to the vigilance state.

## Introduction

The treatment of choice for patients with drug‐resistant epilepsy is epilepsy surgery with the objective of resecting the epileptogenic zone (EZ).^1^ Currently the standard diagnostic work‐up for presurgical epilepsy evaluation consists of video‐electroencephalography (EEG), structural magnetic resonance imaging, and neuropsychology. In recent years there is increasing evidence of the value of electrical source imaging (ESI) in the presurgical evaluation of epilepsy.^2^, ^3^ ESI provides a way to detect the source of scalp EEG potentials in the brain. ESI is performed by solving an inverse problem,^4^ which aims to estimate the most likely source of the observed scalp EEG potentials. There are two main approaches. The first is dipole modeling,^5^ which attempts to identify a single point source or a few point sources in the brain that explain the distribution of scalp EEG potentials. The second approach involves distributed methods,^6^ which place dipoles throughout the cortical surface in alignment with the brain's structure. This approach then attempts to determine each dipole's amplitude contribution in order to explain the distribution of scalp EEG potentials. The advantage of distributed methods is its ability to assess the source extent, that is the boundaries of the active region. In the context of epilepsy, when ESI uses distributed methods and is performed correctly,^7^ it can accurately pinpoint the source of epileptic activity and delineate its extent. To achieve accurate localizations, low noise, and high spatial sampling with high‐density EEG (HD‐EEG)^8^ are preferred.

Increasing evidence from intracranial EEG revealed the relevance of the effect of different states of vigilance on epileptic activity.^9^, ^10^ Interictal epileptic discharges (IEDs) were observed to increase in frequency during non‐rapid eye movement sleep (NREM) and to be suppressed during REM sleep.^11^, ^12^ The state of vigilance was also shown to influence IED morphology and topography, with more circumscribed IEDs occurring during REM as opposed to NREM sleep.^13^, ^14^ In addition, the distribution and type of seizures,^11^ but not their spatio‐temporal dynamics^15^ have been shown to be influenced by the vigilance state. Finally, there is some evidence from ESI that REM sleep may reveal topographically new foci that are not present in other vigilance states,^16^, ^17^, ^18^ although evidence on the latter remains sparse.^19^

A recent review on the topography of IEDs across the different states of vigilance stated that REM IEDs were in better agreement with the clinical SOZ than NREM IEDs (82 vs. 60%).^16^ However, only two studies used ESI, one in a small sample of 6 patients using HD‐EEG^17^ and the other one in 16 patients using low‐resolution 25‐electrode ESI.^18^ Both studies suggested REM ESIs offer unique spatial coordinates and extent when compared to other vigilance states. However, the accuracy of ESI and estimation of the underlying source extent of the generators can vary when considering a source imaging method not designed to recover the extent of the underlying generators of IEDs. In addition, other attributes such as a low‐density array or low numbers of IEDs would also affect ESI accuracy. Therefore, more work is warranted to clarify these findings in larger sample sizes with higher numbers of IEDs during REM sleep and HD‐EEG. This is particularly relevant, as currently ESI is mostly performed with IEDs recorded during the awake state in short‐term recordings. To substantiate evidence that REM may achieve more accurate localization results would have direct impact on our clinical practice.

Source localization is not trivial and a good spatial sampling from scalp recordings is crucial for obtaining a reliable source map.^8^ In addition, source localization methods are known to be influenced by the signal‐to‐noise ratio (SNR),^20^ and assessing the underlying source extent remains a challenging task.^21^, ^22^, ^23^ To address these challenges, one can use HD‐EEG for spatial coverage and a distributed ESI methodology sensitive to source localization and extent. One of these methods is the coherent Maximum Entropy on the Mean (cMEM) method.^24^, ^25^ Our group carefully evaluated the ability of cMEM to recover the source extent of the generators, when compared to other standard distributed source modeling approaches.^21^, ^22^, ^26^, ^27^, ^28^ Applying cMEM localization on manually marked IEDs, recorded using a HD‐EEG array, we were able to produce accurate results in identifying the EZ.^29^

In this study, we compared the IED source localization maps across different vigilance states when applied to HD‐EEG data and using cMEM as an ESI method sensitive to the source extent. We report here that, contrary to our primary hypothesis, IED source localization during REM sleep does not seem to offer a superior localization of IEDs when compared to any other state of vigilance.

## Methods

### Subjects

Twenty‐six consecutive patients (7 females; mean age, 33.56 ± 10.91 years) with a diagnosis of drug‐resistant focal epilepsy who underwent between 24 and 48 h long recordings of combined polysomnography (PSG) and HD‐EEG (83 electrodes) at the Montreal Neurological Institute and Hospital between January 2019 and July 2022 were screened. Patients were consecutively recruited based on the referral of the clinical team for the indication of epilepsy presurgical evaluation, availability of a technician to place all electrodes necessary for the HD‐EEG recording, and absence of prior brain surgery. The presence of IEDs in prior recordings was not a prerequisite to perform HD‐EEG. We excluded 8 patients that did not have a minimum of 5 IEDs in REM sleep (this was the first criterion), one whose recording contained too many artifacts to reliably score sleep, and one patient, whose brain imaging could not be properly segmented due to an extensive encephalomalacic lesion. A total of 16 patients (7 females; mean age, 33.56 ± 10.91 years) were included. Our cohort consisted of 10 temporal (62.5%) and 6 extratemporal patients. A subset of 11 patients underwent surgery, out of which 8 (72%) were classified as seizure‐free (Engel IA) after 23.5 [14–44] months of follow‐up (Table 2). All study participants provided written informed consent in agreement with the Research Ethics Board at the Montreal Neurological Institute (REB00010120). All data were analyzed in an average reference montage.

### HD‐EEG recordings and signal analysis

HD‐EEG recordings were collected during the patients' hospitalization for presurgical evaluation in the epilepsy monitoring unit. Recordings were performed with the Nihon Koden system (Tokyo, Japan) using collodion glued 83 electrodes placed according to the 10–10 EEG system and sampled at 1000 Hz. Electrodes positions together with additional headshape points were digitized on the scalp of the patients using a Polhemus localizer device (Vermont, USA). Then, the electrodes were aligned on the head model (see SI methods) using reference points (nasion, right, and left ear). Coregistration was refined using a surface fitting approach (iterative closest point) using *Brainstorm*. Finally, electrode positions were projected on the scalp surface.

### Marking of IEDs and definition of the clinical ground truth

IEDs were marked by visual inspection of a neurophysiologist in all vigilance states. Not all IEDs were marked, but we marked rather a sufficient amount of IEDs providing good SNR with a minimum of 5 IEDs in each state of vigilance of the predominant IED type as done in our previous work.^30^ This was done in order to propose an analysis similar to the one considered for clinical purposes, in which not all available IEDs are marked but only the ones that are deemed to have high quality. This is important as ESI is affected by the quality, the amount, and SNR of the final average IED which is being localized. IEDs during wakefulness were marked prior to the night sleep recording. The IEDs were averaged ±5 s around the negative peak of the IED, then the midpoint of the average IED, defined as half way of the ascending slope of the IED, was marked by visual inspection, and confirmed by a neurophysiologist. This midpoint was considered when evaluating ESI maps. There is debate in the literature about the point of the averaged IED that should be used when calculating the ESI, with some authors advocating for the point at 50% of the rising phase^3^ or the take‐off,^31^ while others show no differences between this point (50% of the rising phase) and the peak of the IED.^32^ We chose the midpoint as a comprise between a good SNR and early IED propagations.

The clinical ground truth was defined by epileptologists (C.A., B.F.) based on Phase 1 or 2 presurgical evaluation with long‐term video EEG monitoring, anatomical 3T MRI, positron emission tomography, and neuropsychological evaluation, or stereo‐electroencephalography where applicable. This ground truth was drawn as a region of interest (ROI) along the cortical surface on each patient's head model. This ROI was then used as the ground truth for all comparisons.

### Electrical source imaging and evaluation of different vigilance states

**Figure 1:**
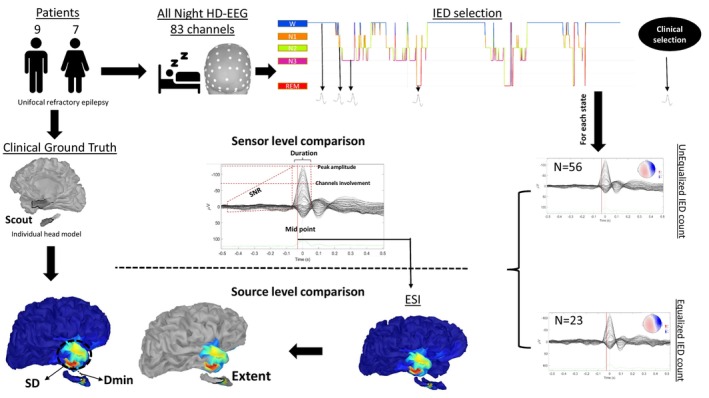
Study workflow. The study used all‐night high‐density electroencephalography (HD‐EEG) recordings to assess the sensor (at IED peak) and source space (at midpoint) influence of the different vigilance states, and compared them to the clinically selected IEDs as well as to the clinical ground truth in the source space. Dmin, distance minimum; ESI, electrical source imaging; IED, interictal epileptic discharge; SD, spatial dispersion; SNR, signal‐to‐noise ratio.

For each patient, the averaged IED was assessed for the four vigilance states (N2, N3, REM, Wake) as well as compared to the IEDs chosen for ESI done in clinical routine (clinical IEDs) which were selected on the bases of good visual SNR. This assessment was performed both in the sensor space (see SI methods) at the peak of the averaged IED as well as in the source space at the midpoint of the averaged IED (Fig. 1). The sensor space refers to the EEG representations observed on a standard tracing at the scalp level, while the source space pertains to the outcomes of the ESI, mapping the brain's sources of these scalp tracings. The peak was chosen as the best representation of the sensor space as viewed by an electrophysiologist, while the midpoint of the IED was chosen for the source space, in order to represent a compromise between SNR and propagation.

The ESI maps were calculated using a depth‐weighted version of cMEM (see SI methods).^33^ The inverse problem was solved using the maximum entropy of the mean (MEM) framework.^24^ The coherent MEM's (cMEM) prior distribution uses a hidden variable associated to each parcel which is tuning the probability of the parcel to be active or to be switched off. Using such a flexible prior distribution, the cMEM method is able to recover accurately the source extent of the underlying generators.^22^, ^26^ The ESI was done between ±50 ms around the peak of the interictal epileptic discharge, but only the ESI results at the midpoint of the averaged IEDs were reported. We proposed two‐level assessments of our evaluation metrics: first by considering all marked IEDs in each state (unequalized condition), second by randomly selecting IEDs in equal proportion to the number of IEDs in the state with the least available amount (equalized condition). The latter was performed in order to address the fact that some vigilance states, such as N3 sleep state, usually have higher IED rates, while minimizing the SNR effect on signal averaging when assessing source localization results. Our proposed approach allows us to investigate both the “real world” clinical situation in which this discrepancy indeed exists, as well as to test whether the final results are simply driven by SNR discrepancies. We evaluated the sensor level using the following metrics: (1) peak amplitude, (2) IED duration, (3) SNR, and (4) channel involvement. We then evaluated the source level and assessed (1) minimum distance (Dmin), (2) spatial dispersion, (3) D‐maxima, (4) source extent, and (5) spatial profile. All metrics are provided and defined in Table 1 and in the SI methods section. Finally, we also performed a sub‐lobar clinical analysis of the ESI maps to determine the sub‐lobar concordance of the maps in the various vigilance states.

**Table 1:** Metrics and definitions employed in the paper to define terms related to both sensor and source space analysis.

	Definition	Interpretation	Space
Peak amplitude (mv)	The peak negative amplitude of the averaged IED's most negative channel.	The peak level at which the IED appears on the EEG trace. A higher number indicates a stronger electrical potential associated with the IED.	Sensor
IED Duration (ms)	The duration from the start to the end of the averaged IED.	A higher number indicates a wider IED.	Sensor
Signal‐to‐noise ratio	The difference between the IED's peak and its background.	A higher number signifies that the IED stands out more from its surroundings. A value of one indicates the noise level.	Sensor
Channel involvement	The number of channels exceeding a specific amplitude threshold during the peak of the IED.	The higher the number of involved channels, the larger is the field spread on the scalp EEG.	Sensor
Minimum distance (mm)	The minimum Euclidian distance between the maximum point of the ESI map to the ground truth ROI.	Reflects how good the ESI is in relation to the ground truth, such that if the maximum of the ESI is within the ground truth the value is zero.	Source
Spatial dispersion (mm)	The quantity and strength of activated areas in the ESI that lie outside the ground truth's ROI.	A smaller number indicates better localization quality relative to the ground truth. A value of zero signifies that all activity was confined within the ground truth's ROI.	Source
D‐maxima (mm)	The distance between the peak points of the ESI maps from two different vigilance states.	A lower number indicates that the peaks of the ESI maps are closer together in space.	Source
Source extent	The proportion of vertices in the ESI map that are marked as active beyond a certain amplitude threshold.	A higher value indicates a more widespread ESI map.	Source
Spatial profile	The spatial distribution of amplitudes in different areas of the brain.	Examining the clustering of the spatial profiles of ESI maps helps to evaluate the overall similarity between maps. A value of one indicates identical maps.	Source

EEG, electroencephalogram; ESI, electrical source imaging; IED, interictal epileptiform discharge; ROI, region of interest.

### Statistical analysis

We assessed evaluation metrics with ANOVA and Tukey or Friedman and Nemenyi tests, depending on distribution. The Shapiro–Wilk test checked distribution normality. *P*‐values were corrected for multiple comparisons using the false discovery rate with alpha set to 0.05. Effect sizes were calculated with Cohen's d, Eta squared, Cliff's *d*, and Kendall's W. Metrics followed either normal (mean, standard deviation) or non‐normal (median, interquartile ranges) distributions. Results are reported for both conditions (unequalized, equalized IEDs). We also compared two hierarchical clustering approaches using a chi‐squared test to classify every single IED source localization result in three classes, NREM, REM, and wakefulness. A paired *t*‐test assessed chi‐squared differences between clusters with/without amplitude information (see SI methods).

## Results

### IED characteristics vary across the different vigilance states

We analyzed a total of 3449 IEDs in 16 patients (Table 2). We analyzed the average IED in each vigilance state as well as the clinically selected average IED, first in the sensor space and later in the source space (Table 3). In the sensor space we found that the number of IEDs, reflecting their discoverability on the scalp EEG, was affected by the vigilance state (*F* (4, 16) = 23.89, *P* < 0.001) with N3 exhibiting the highest numbers. Furthermore, we observed an effect of the vigilance state on the IED amplitude for both the unequalized and the equalized conditions (*F* (4, 16) = 21.1, 19.65; *P* < 0.001, <0.001; *w* = 0.33, 0.31, respectively). The average unequalized and equalized IED peak amplitude during N3 was overall larger when compared to REM (*P* = 0.004, 0.002; *d* = 0.84, 0.78) and wakefulness conditions (*P* = 0.005, 0.002; *d* = 0.82, 0.80) (Fig. 2A). A similar observation was found between N2 and REM (*P* = 0.007, 0.005; *d* = 0.76, 0.72), as well as between the clinically selected IEDs and REM (*P* = 0.007, 0.002; *d* = 0.75, 0.72). The IED duration from take‐off to take‐down was also affected by the state of vigilance in both conditions (*F* (15, 60) = 13.22, 10.17; *P* < 0.001, <0.001; ŋ = 0.72, 0.68). The duration in N3 was longer (Fig. 2B) when compared to REM (*P* = 0.07, 0.03; *d* = 0.65, 0.74), and wakefulness (*P* = 0.02, 0.03; *d* = 0.9, 0.8). The SNR of the unequalized condition was similarly affected by the vigilance state (F (4, 16) = 23.05, *P* < 0.001, *w* = 0.36). N3 and the clinically selected average IED were exhibiting a higher SNR (Fig. 2C) when compared to REM (N3‐R: *P* < 0.001, *d* = 0.65; clinical‐R: *P* < 0.001, *d* = 0.81) and wakefulness (N3‐W: *P* < 0.001, *d* = 0.56; clinical‐W: *d* = 0.85, *P* < 0.001). When considering the same number of IEDs in each condition, no significant differences in SNR were observed between any states (*P* > 0.05).

**Table 2:** Patient's demographics.

#	Age at HD‐EEG	Sex	Age at seizure‐onset	MRI	SEEG	Ground truth (sub‐lobar level)	Surgery/Engel^1^
1	40	M	25	Normal	Yes (IEDs: L amygdala, hippocampus, entorhinal cortex, anterior fusiform; SOZ: L amygdala, hippocampus, entorhinal cortex)	L MTLE	Yes/IIIb (2 years)
2	32	M	18	R temporal neocortex atrophy, atrophy/agenesis of the R piriform	Yes (IEDs: R posterior insula, posterior temporal gyrus; SOZ: R posterior temporo‐insular junction)	R posterior temporo‐insular	Yes/Ia (>1 year)
3	27	F	5	Normal	Yes (IEDs: R superior, middle, inferior occipital gyri; SOZ: R superior and middle gyri)	R latero‐occipital	Yes/Ib (>1 year)
4	39	M	25	Right hippocampal sclerosis	No	R MTLE	No
5	39	F	12	Right hippocampal sclerosis	No	R MTLE	Yes/Ia (10 months)
6	26	F	16	Normal	Yes (IEDs: L hippocampus, amygdala, and entorhinal cortex; SOZ: L hippocampus, amygdala, and entorhinal cortex)	L MTLE	Yes/Ia (>4 years)
7	41	F	16	Right hippocampal sclerosis	No	R MTLE	Yes/Ia (>3 years)
8	51	F	43	R hippocampal atrophy	No	R MTLE	Yes/Ia (>3 years)
9	23	M	16	R posterior insula FCD	No	R parieto‐operculo‐insular junction	No
10	43	M	29	Possible R hippocampal sclerosis	No	R MTLE	Yes/Ia (>1 year)
11	19	F	6	R lingual encephalomalacic lesion	No	R mesio‐occipital	Yes/IV (>1 year)
12	20	M	11	R mesial premotor FCD	No	R mesial premotor	No
13	51	M	49	Bilateral mesiotemporal abnormalities (L FCD, R atrophy)	No	L MTLE	No
14	18	F	9	Normal	Yes (IEDs: R posterior fusiform, lingual gyrus; SOZ: no spontaneous seizure recorded)	R mesial temporo‐occipital	Yes/Ia (>2 years)
15	24	M	23	R fronto‐polar FCD	No	R fronto‐polar	No
16	44	M	Early childhood	L hippocampal sclerosis	No	L MTLE	Yes/III (>1 year)

L, left; R, right, MTLE, mesio‐temporal lobe epilepsy; FCD, focal cortical dysplasia.

^1^ Follow‐up >1 year after surgery.

**Table 3:** Interictal epileptic discharges count for every vigilance state, and the corresponding sub‐lobar results of the electrical source imaging map.

	# IEDs					Anatomical location (sub‐lobar source localization)				
#	N2	N3	R	W	C	N2	N3	R	W	C
1	58	19	7	19	30	L temporal pole	L temporal pole	L temporal pole	L temporal pole	L temporal pole
2	22	25	12	5	114	R posterior & lateral temporal	R posterior temporo‐insular	R posterior temporo‐insular	R posterior & lateral temporal	R posterior temporo‐insular
3	23	24	35	24	223	R posterior & lateral temporal	R posterior & lateral temporal	R posterior & lateral temporal	R posterior & lateral temporal	R posterior & lateral temporal
4	28	27	54	36	92	R temporal pole	R temporal pole	R temporal pole	R temporal pole	R temporal pole
5	24	24	34	23	56	R anterior & lateral temporal	R anterior & lateral temporal	R temporal pole	R anterior & lateral temporal	R anterior & lateral temporal
6	33	25	35	27	24	L mesio‐temporal	L mesio‐temporal	L mesio‐temporal	L mesio‐temporal	L mesio‐temporal
7	35	74	13	8	88	R anterior & lateral temporal	R anterior & lateral temporal	R anterior & basal temporal	R anterior & lateral temporal	R anterior & lateral temporal
8	19	69	9	6	73	R temporal pole	R temporal pole	R temporal pole	R anterior & lateral temporal	R temporal pole
9	24	18	30	30	128	R central	R lateral parietal	R lateral parietal	R posterior & basal temporal	R anterior & basal temporal
10	15	226	7	6	76	R temporal pole	R temporal pole	R mesio‐temporal	R temporal pole	R temporal pole
11	37	48	16	10	86	R temporal pole	R anterior & lateral temporal	R temporal pole	R anterior & basal temporal	R temporal pole
12	21	39	16	24	113	R mesial premotor	R mesial premotor	R central	R central	R central
13	11	17	6	8	42	L anterior & lateral temporal	L temporal pole	L temporal pole	L temporal pole	L anterior & basal temporal
14	102	187	55	77	177	R mesial temporo‐occipital	R mesial temporo‐occipital	R mesial temporo‐occipital	R mesial temporo‐occipital	R mesial temporo‐occipital
15	30	17	10	9	18	R dorsolateral prefrontal	R dorsolateral prefrontal	R dorsolateral prefrontal	R dorsolateral prefrontal	R dorsolateral prefrontal
16	6	48	31	8	44	L temporal pole	L temporal pole	L temporal pole	L temporal pole	L temporal pole

**Figure 2:**
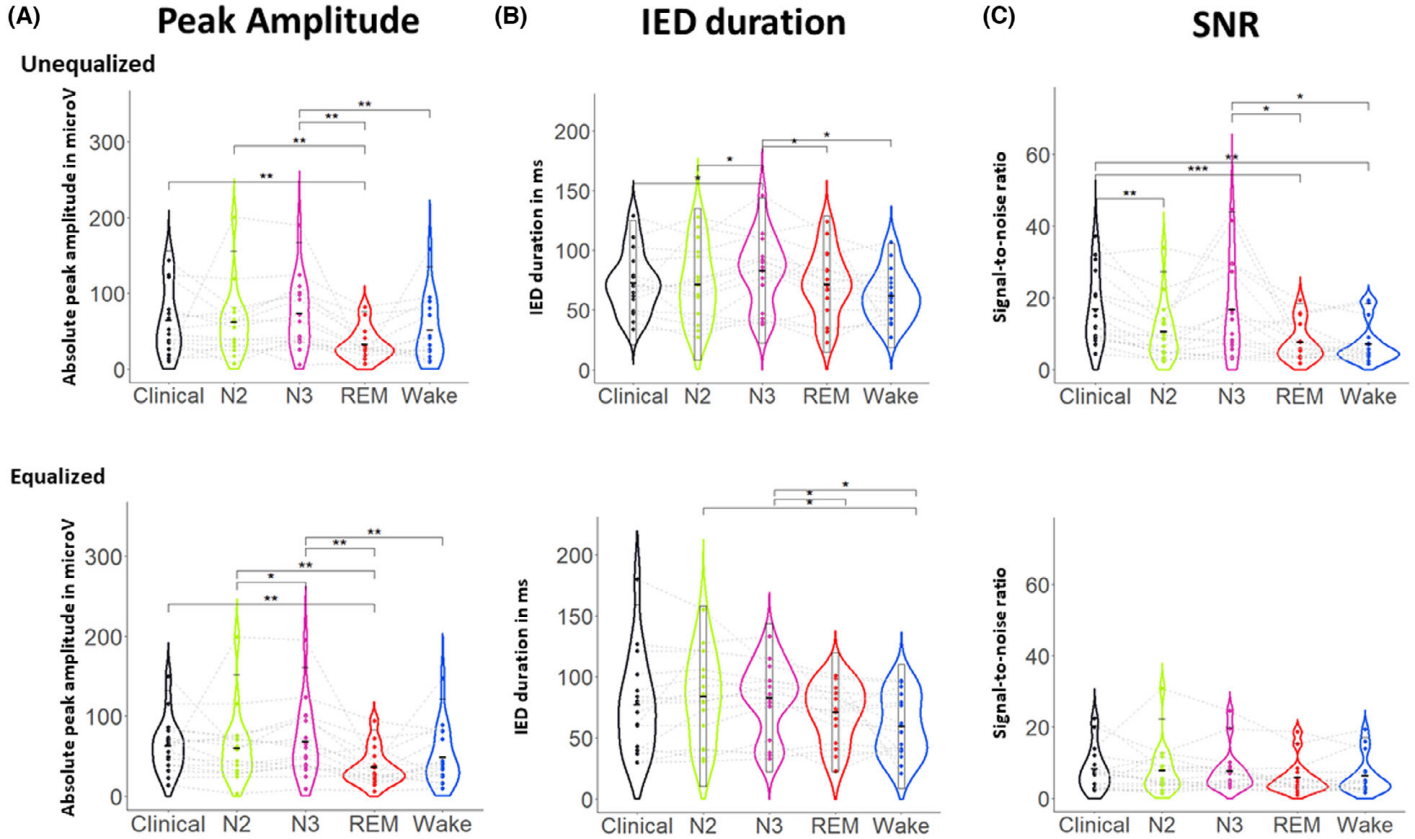
Interictal epileptic discharge (IED) characteristics in different states of vigilance. Distributions of average IED sensor space evaluation metrics for each vigilance state N2, N3, REM, Wake, and clinically selected IEDs, represented as violin plots. (A) amplitude of the IED peak in μV, (B) IED duration in ms, (C) signal‐to‐noise ratio measured on the peak channel, for unequalized (top row) and equalized conditions (bottom row). The lines connecting the dots are linking each patient through all the vigilance states. Significance level were notes as *<0.05, **<0.01, ***<0.001.

### IED sensor involvement varies between different vigilance states

**Figure 3:**
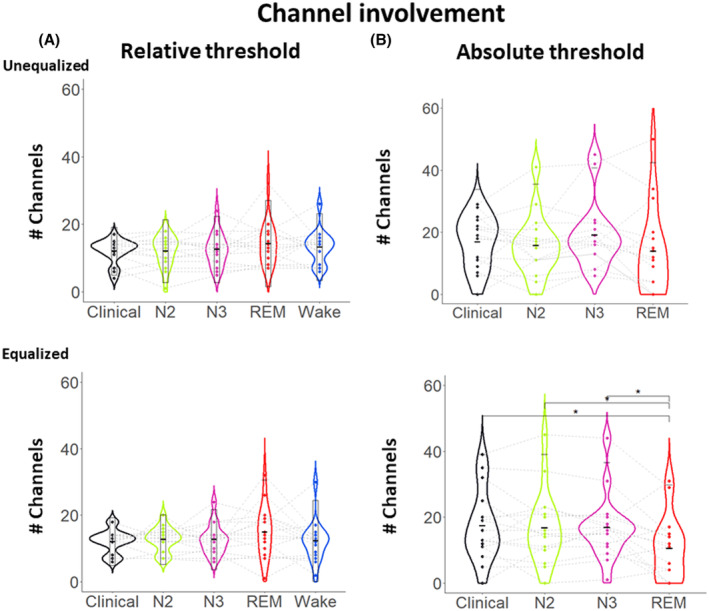
Sensor space variation between vigilance states. Averaged IED channel involvement in different states of vigilance as measured by an amplitude threshold (A) relative channel involvement defined as channels with amplitude >50% of the maximum peak within each state, (B) absolute channel involvement defined as channels with amplitude >50 of the maximum peak of the wakefulness average IED. The lines connecting the dots are linking each patient through all the vigilance states. Significance level were notes as *<0.05, **<0.01, ***<0.001.

Here we examined whether the number of channels that are involved during an IED varies between states. To do so, we counted the number of channels in which the amplitude during the IED peak reached at least 20% of the amplitude of the highest amplitude channel (relative). We found that there were no statistical differences between the different vigilance states (Fig. 3A, *P* > 0.05). We then assessed whether there was an absolute difference rather than a relative one between the states. To test this, we used an absolute threshold (20% of the maximum amplitude of the average IED during wakefulness) for comparisons between states. With this approach, we found that there was a significant effect for both equalized and unequalized conditions (*F* (3, 16) = 8.74, 10.0; *P* = 0.01, 0.032; *w* = 0.21, 0.18); however, during the post hoc analysis, no statistical difference was found after correction for multiple comparisons or correction for the unequalized condition. The equalized condition displayed a lower channel count involved during IEDs in REM (Fig. 3B) when compared to IEDs in N2 (*P* = 0.04; *d* = 0.58), N3 (*P* = 0.04; *d* = 0.63).

### Localization accuracy of IEDs does not vary across different vigilance states

**Figure 4:**
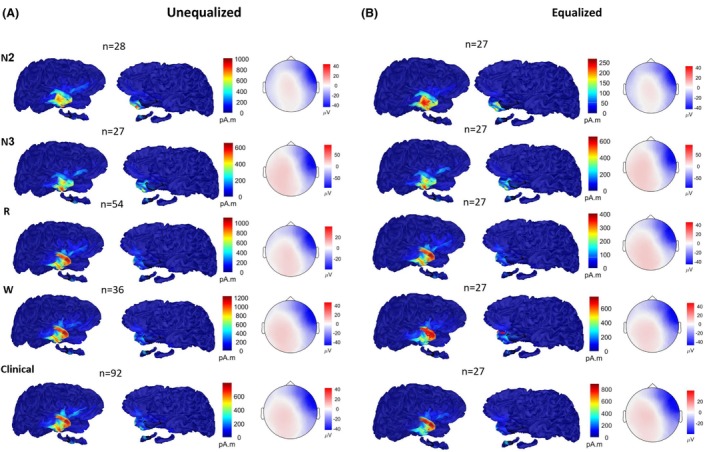
Patient example #3. Source localization maps estimated and voltage maps at the midpoint of average IEDs for all vigilance states (N2, N3, REM, Wake) and for the clinically chosen IEDs, when considering: (A) all available IEDs, (B) equalized number of IEDs. In this case, 27 was the number of IEDs marked during N3 sleep. Note that the scales differ between source maps.

**Figure 5:**
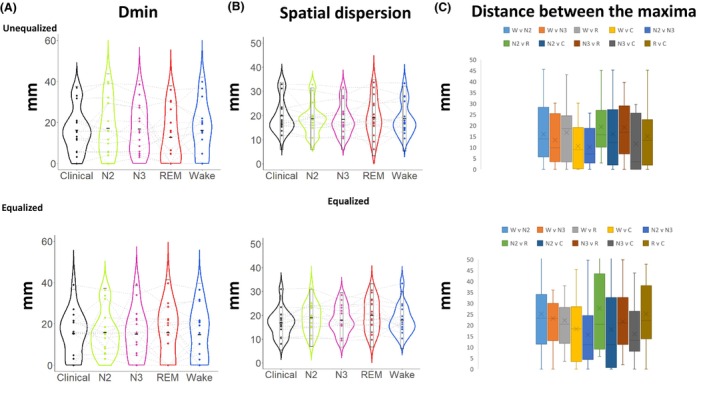
Accuracy of ESI maps in different states. The source localization maps were compared to a clinical ROI marking the presumed epileptogenic zone. (A) Distance minimum (Dmin): measures the distance between the maximum vertex to the scout, (B) spatial dispersion: measures the spatial spread (in mm) of the localization around the ground truth, (C) D‐maxima: measures the distance between each pair of maps maximum vertex in all states of vigilance.

To assess the localization accuracy across different states, in relation to its ability to correctly point and delineate the ROI of the ground truth, we used the ESI source maps estimated at the midpoint of the average IEDs in each state (Fig. 4). We found no significant difference between the vigilance states in either condition (i.e. equalized or unequalized number of IEDs averaged), when assessing the minimum distance from the maximum ESI map activation to the clinical ROI (Fig. 5A, all *P* > 0.05). In addition, no significant difference was observed for accuracy (Fig. 5B) of the localization as reflected in the spatial dispersion, measuring the spatial spread of the ESI map around the clinical ROI (all *P* > 0.05). Finally, we found no significant differences when comparing distances between ESI maps maxima in different vigilance states (Fig. 5C), yet a tendency of N2‐N3 localizations to be closer was observed in both states (all *P* > 0.05). The localization classification on a sub‐lobar level (Table S1) also demonstrated the maps' similarities between different ESI maps (see Fig. 4), where sub‐lobar concordance between the clinical ground truth and the ESI map exhibiting the largest activity was found for 15 out of 16 patients in both conditions in all vigilances states.

### Effect of the vigilance state on the ESI source extent

**Figure 6:**
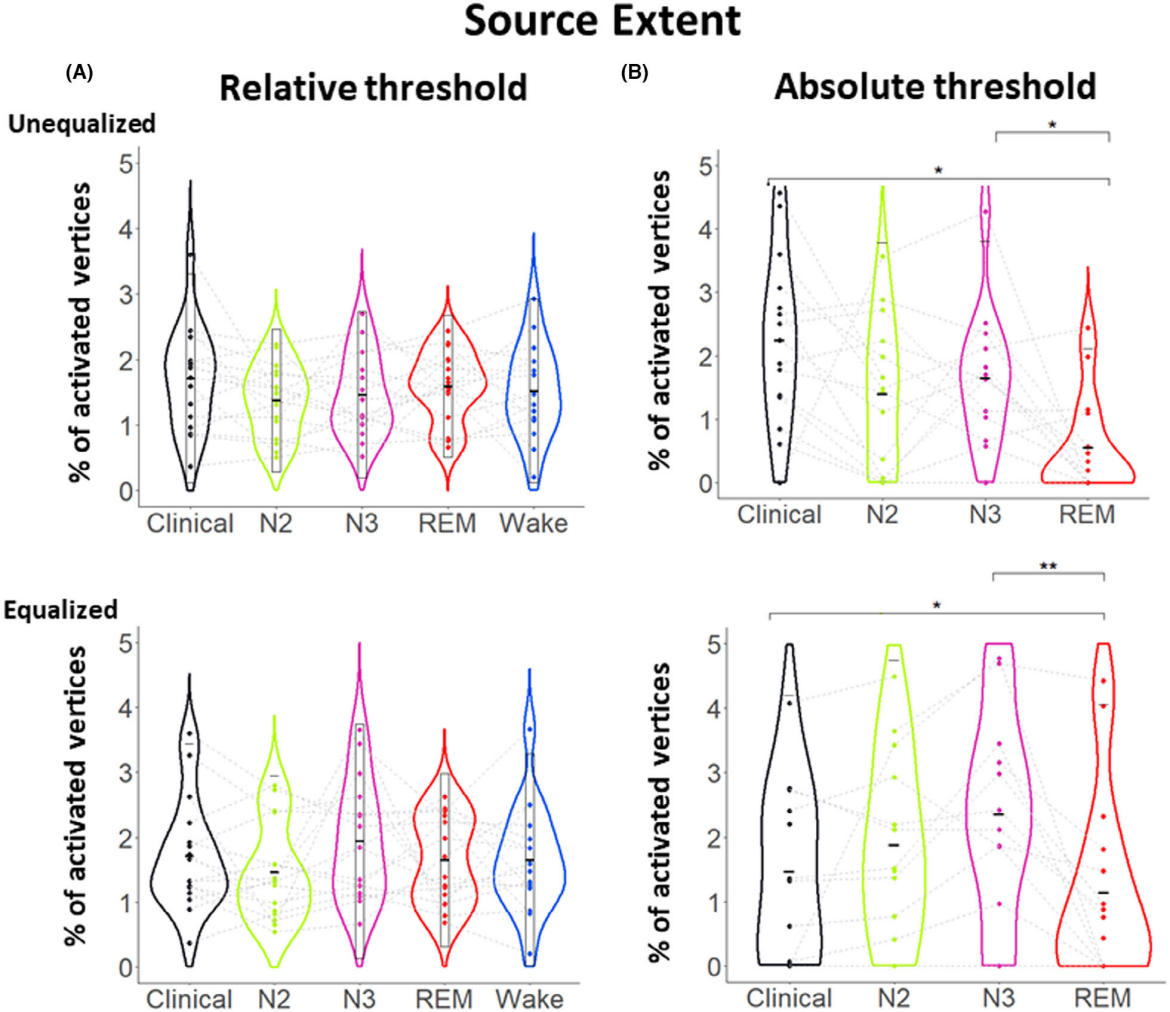
Source space variation between vigilance states. Source extent of average IEDs source maps in different states of vigilance after applying an amplitude threshold in the source space: (A) Relative threshold: percentage of activated vertices >20% of the maximum vertex within each specific state (B) Absolute threshold: percentage of activated vertices >20% of the maximum vertex in the wakefulness average map, considered as reference.

In this section, we investigated the impact of the vigilance state on the source extent of the IED generators in the source space after applying ESI using cMEM. We found that the source extent as expressed by the percentage of activated vertices did not differ between the states when we considered a relative threshold to estimate the extent (Fig. 6A) for both unequalized and equalized conditions (All *P* > 0.05). Similarly, to prior investigation at the sensor level (Fig. 3), we also investigated the source extent when considering the same absolute threshold for all maps, therefore mimicking the clinical investigation. We considered the absolute threshold as 20% of the wakefulness ESI source map. In this condition, we found a significant effect of the vigilance state on the source extent (Fig. 6B) in both the unequalized and equalized conditions (*F* (3, 16) = 12.32, 14.73; *P* = 0.001, 0.004; *w* = 0.26, 0.3). The ESI map during REM was exhibiting a lower percentage of active vertices, that is, a smaller source extent when compared to N3 (*P* = 0.02, 0.008; *d* = 0.67, 0.82) and to the clinically proposed solution (*P* = 0.02, 0.03; *d* = 0.64, 0.75), therefore suggesting a more focal involvement of the IED generators during REM.

**Figure 7:**
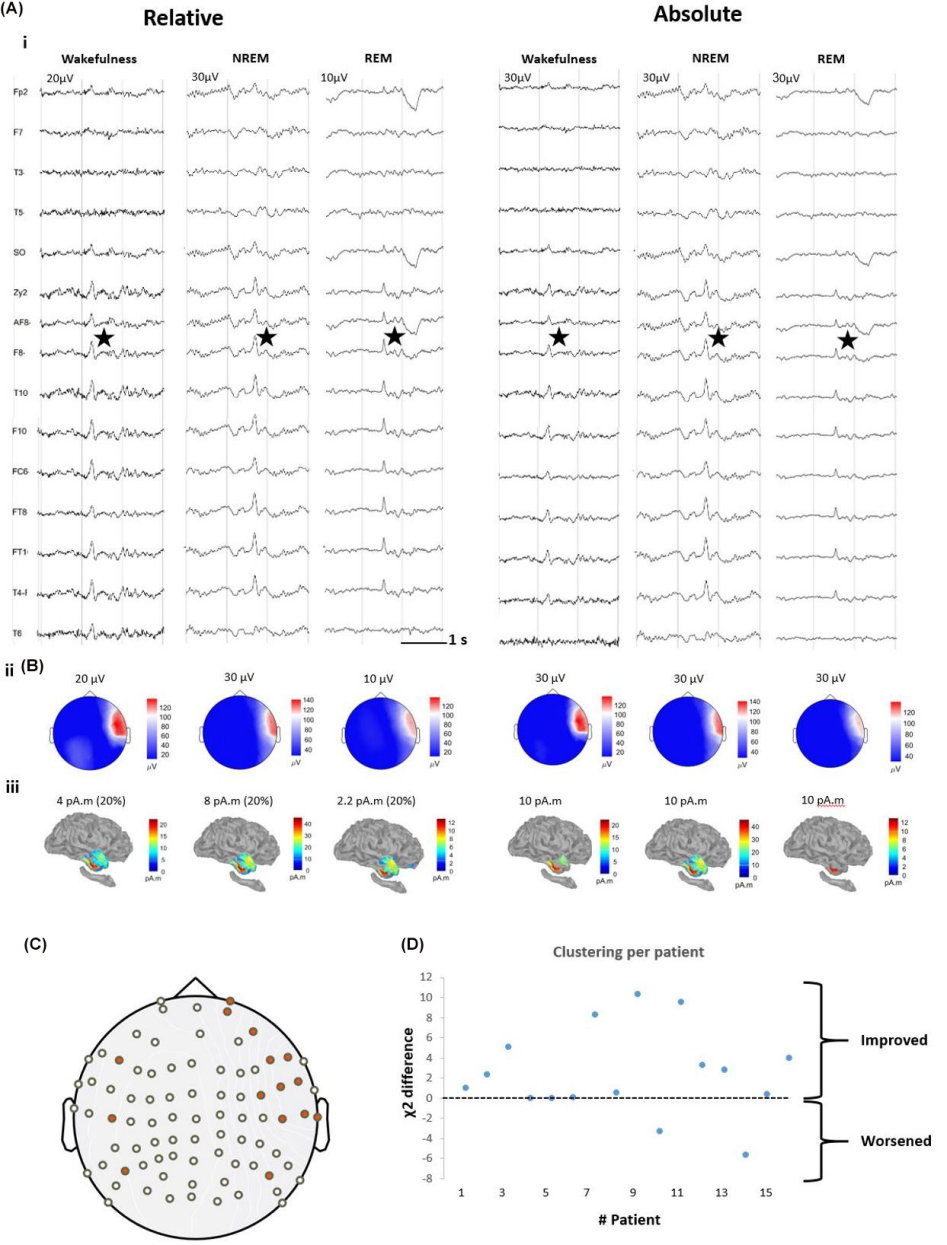
The effect of relative and absolute threshold in the sensor and source space (A) Spatial distribution examples of IEDs during NREM, REM, and Wake from patient #5, who is a 39‐year‐old female with drug‐resistant right mesiotemporal lobe epilepsy. (B) (i) The interictal EEG showed IEDs predominantly over the right anterior to mid‐temporal region (maximum amplitude over F8, FT8, FT10, T4, and Zy2), (ii) the corresponding voltage map, (iii) the corresponding source maps of the IEDs, (C) 2‐D view of the high‐density EEG with the selected electrodes in red which were selected in panel A. (D) Clustering differences with and without amplitude information. Differences in chi‐squared results for all patients, with positive values indicating that the clustering improved differentiation between the different states of vigilance when including amplitude information in the clustering.

Finally, we investigated whether the difference in the source extent observed during REM sleep was mainly driven by amplitude differences, as the discrepancy between relative and absolute thresholds results suggested (Fig. 7A–C). To examine these spatial profiles, we performed a clustering analysis after applying cMEM on all available single IEDs in each vigilance state. We proposed two data‐driven clustering approaches of all those ESI maps, one including and one not including ESI amplitude information (see SI methods). Each clustering resulted in three classes, and such a classification was compared with the true vigilance state labeling (Awake, NREM, REM) using a chi‐squared metric. We found that amplitude‐sensitive clustering (*χ* ^2^ = 15.96 ± 9.42) was exhibiting a more skewed distribution (Fig. 7D) when compared to the amplitude‐invariant one which only uses the shape of ESI distribution while omitting all information related to amplitude (*χ* ^2^ = 13.54 ± 9.47; *P* = 0.03; *d* = 0.24). These results are supporting the idea that for most patients the amplitude of the ESI maps aided in distinguishing between the maps in different vigilance states, rather than changes in the spatial distribution of the sources themselves.

## Discussion

In recent years the effect of the vigilance state on the ability to correctly localize the EZ received increasing attention,^9^, ^17^ with some authors suggesting that the state of vigilance can alter the results of ESI's spatial profile. Confirmation of this hypothesis has the potential to change how we perform ESI in clinical practice. Here, we aimed to understand the influence of the vigilance state on the ability to accurately identify the sources of IEDs. We compared the localizability of IEDs during different vigilance states against two gold standards, the set of clinically selected IEDs (i.e. when not taking into account the vigilance state) and the clinical ground truth defined by clinical judgment based on all presurgical information. Our overall findings indicate that (i) NREM is associated with higher IED numbers, higher amplitude peaks, and longer duration, but that (ii) localizability of IEDs is not significantly impacted by the vigilance state; and that (iii) the effect of the state of vigilance on IED localizability is primarily driven by amplitude and IED numbers rather than the state of vigilance itself. Indeed, the spatial profile of the IED sources was not significantly influenced by the vigilance state when controlled for IED amplitude. Overall, in contrary to our primary hypothesis, our results suggest that the state of vigilance may hence not be a crucial factor for IED source localization.

### The state of vigilance does not significantly impact the results of ESI

ESI is becoming a useful tool in presurgical evaluation of epilepsy patients,^2^, ^34^ However, if an interaction between the vigilance state and ESI results exists, it has the potential to impact patient care by either hindering or improving the accuracy of ESI results. We indeed found a high consistency between the various vigilance states and the clinical ground truth (Fig. 5). ESI studies in the field of sleep and IEDs are scarce. To date, only two studies have been conducted, one using a low‐density array with 25 channels,^18^ and the other using a high‐density array with 256 channels.^17^ Our study supports the findings of the first study of McLeod et al,^18^ which found no difference in source extent between different vigilance states except when comparing the extent during REM sleep between unifocal and multifocal patients. However, since our study only included unifocal patients, we cannot confirm this observation. Furthermore, this study reported that REM had the most discordance with other vigilance states. However, in our analysis, we did not find any significant differences when using the maximum localization distances between different vigilance states. This discrepancy may be due to differences in the methodological approaches. The second study by Kang et al^17^ reported that ESI localizations were exhibiting fewer vertices involved during REM when compared to NREM. Our results also support this claim, but only when we considered an absolute threshold defined using the awake state for reference (as shown in Figs. 3B and 6B). Indeed, in our study, we carefully demonstrated that the little effect on ESI accuracy in different vigilance states was mainly driven by ESI amplitude and not by the spatial profile.

### Source localization as a measure of source extent

ESI can be challenging as it can vary depending on factors such as the density of the array,^35^ the SNR,^20^ or the methodology^27^ used. Assessing the source extent of the sources can be even more difficult, as different methods can result in different findings,^36^ and most standard linear approaches are indeed not sensitive to the underlying source extent.^21^, ^22^, ^28^ This is particularly problematic in cases of deep epileptic sources, as deep sources are traditionally difficult to detect using ESI.^37^ Given these limitations and our interest in assessing accurately the underlying source extent of the generators, we considered an updated version of cMEM that has been shown to accurately address this matter.^22^, ^27^ While relying on cMEM's ability to recover the source extent of the generators, here we adapted a depth‐weighting implementation of cMEM,^38^ allowing deeper sources to be amplified and assessed more accurately. We used this method to allow inclusion of mesiotemporal lobe epilepsy cases in our cohort. For the same reason, we also included a surface segmentation of both hippocampi in the source space, these were modeled as distributed dipolar sources perpendicular to this hippocampus surface.^39^ A previous HD‐EEG ESI study compared MNE and cMEM in different vigilance states.^17^ Interestingly, the authors found that the effect of the vigilance state on the extent was reduced when cMEM was used reaching only borderline significance (*P* = 0.04). This discrepancy may explain our results, as we analyzed more patients (16 vs. 6) and higher IED numbers using cMEM which would result in more spatially reliable source maps.

### Clinical quality of ESI is more impacted by amplitude and number of IEDs than the state of vigilance

The quality and spatial accuracy of ESI in relation to the clinical ground truth, as shown by spatial dispersion, assessing how much the ESI map was spreading around the clinical ROI, was more sensitive to the number of IEDs than to the vigilance state. An increase in spatial dispersion was noted in the equalized condition, when a lower number of IEDs were averaged (Fig. 5B), with no difference observed between vigilance states. Additionally, while no significant variation was found in the maxima distances between the different vigilance states, the overall D‐maxima distance ranges also increased in the equalized condition, that is, when a lower number of IEDs was averaged (Fig. 5C). Interestingly, the distance of the maximum of the map from the ground truth Dmin appeared unaffected (Fig. 5A). This highlights the crucial role of the number of IEDs, in determining the reliability of the ESI extent. This important issue might have impacted previous studies which explored the extent of the ESI using a low number of IEDs,^17^, ^18^ and more likely lower SNR data. Of the initial cohort in our study, 8 patients (30%) did not reach the minimum of 5 IEDs during REM sleep, which we chose as an arbitrary criterion to achieve a reliable source localization. Our findings also align with previous research^16^ indicating that these characteristics are typically different between the various stages of vigilance with the largest differences being observed between NREM and REM sleep. However, there is some heterogeneity of the definition of the clinical ground truth at the individual level. Eleven patients out of 16 underwent surgery (5 had SEEG prior to surgery). Eight are Engel I and 3 are Engel II‐IV. For the 5 remaining patients, 1 had hippocampal sclerosis and 4 had focal cortical dysplasia type II lesions visible on the MRI. lt is important to mention that despite this heterogeneity of the definition of the ground truth, a great concordance between the MRI lesion, the semiology, the video‐EEG monitoring as well as the high‐density EEG, and the neuropsychological findings was observed in all 5 patients who did not yet have surgery.

### Choice of the threshold matters for IED and source extent

Previous studies have suggested that the channel involvement^17^ and source extent^17^, ^18^ may be more focal during REM sleep. However, our analysis using relative thresholds found no significant difference. Yet, when using an absolute threshold defined considering data in the awake state, we did observe an effect and found that REM sleep scalp data and localizations were exhibiting fewer involved channels and vertices (Figs. 3B and 6B), aligning with these of the previous investigations. The reoccurrence of the same discrepancy in the extent between relative and absolute threshold in both sensor (Fig. 3) and source space (Fig. 6) raises the question of what drives the source extent in those results. Our results showed that incorporating amplitude information improved the clustering results (Fig. 7), indicating that the amplitude itself contains valuable information, rather than just the source distribution, to classify ESI maps to each specific vigilance state. This could also explain the discrepancies observed in previous studies which did not consider this factor.^17^, ^18^

### Limitations

This study utilized a moderate‐sized epilepsy cohort, as we needed to exclude 30% of patients due to stringent criteria of minimum 5 IEDs per vigilance state. Despite achieving source assessment with Engel Class 1 outcomes only in patients undergoing surgery during recruitment, we leveraged the clinical ground truth to mitigate this limitation. To ensure clinical relevance beyond statistical significance, we calculated effect sizes and compared sub‐lobar level results. Finally, not all ground truths are equivalent in our study. A perfect ground truth of an Engel 1A outcome was only available in 8 patients.

## Conclusion

The results of this study did not support the hypothesis that REM sleep results in ESI maps that are more focal or topographically distinct. This attests to the reliability of ESI across the different vigilance states. Our findings also demonstrated that amplitude and the number of IEDs is indeed more important than the state of vigilance.

## Conflicts of Interest

None of the authors has any conflict of interest to disclose. Outside of this work, BF received speaker / advisory board honoraria from Eisai, Paladin labs, UCB, and UNEEG.

## Supporting information


**Methods S1.** Contains an explanation of the methods used including a full description of the source localization methodology and precise functions of the metrics used.
**Table S1.** All the values measured for each metric for every condition at every sleep stage.
