# Supplementary material for: Consistency of electrical source imaging in presurgical evaluation of epilepsy across different vigilance states
Source: Ann Clin Transl Neurol. 2024 Jan 12;11(2):389–403. doi: 10.1002/acn3.51959 (PMC10863930; doi:10.1002/acn3.51959)
Supplement: Supplementary file 1 — Methods S1. Contains an explanation of the methods used including a full description of the source localization methodology and precise functions of the metrics used. Table S1. All the values measured for each metric for every condition at every sleep stage. [file ACN3-11-389-s001.docx]

**Supporting Information**

**Consistency of electrical source imaging in presurgical evaluation of epilepsy across different vigilance states**

**Author affiliations:**

Tamir Avigdor*^a,b^, Chifaou Abdallah*^a,b^, Jawata Afnan^b^, Zhengchen Cai ^c^, Saba Rammal^a^, Christophe Grova^b,d#^, Birgit Frauscher^a,e,f#^

a. Analytical Neurophysiology Lab, Montreal Neurological Institute and Hospital, McGill University, Montreal, Quebec, Canada

b. Multimodal Functional Imaging Lab, Biomedical Engineering Department, McGill University, Canada

c. Montreal Neurological Institute and Hospital, McGill University, Montreal, Quebec, Canada

d. Multimodal Functional Imaging Lab, PERFORM Centre, Department of Physics, Concordia University, Montreal, QC, Canada

e. Department of Neurology, Duke University Medical Center, Durham, North Carolina, USA

f. Department of Biomedical Engineering. Duke Pratt School of Engineering. Durham, North Carolina, USA

* Both authors contributed equally to this manuscript.

# shared senior authors

**Corresponding author:**

Birgit Frauscher, Analytical Neurophysiology Lab, Department of Neurology, Duke University Medical Center, Hock Plaza, 2424 Erwin Road, Durham, NC, 27705, USA. [birgit.frauscher@duke.edu](mailto:birgit.frauscher@duke.edu)

**Acknowledgments**

We wish to express our gratitude to the staff and technicians at the EEG Department of the Montreal Neurological Institute and Hospital, particularly Erica Minato, Lorraine Allard, Nicole Drouin and Chantal Lessard.

**Funding:** This work was funded by project grants from the Canadian Institutes of Health Research (PJT-175056 to B.F., PJT 159948 to C.G). The study was supported by the Savoy Foundation Fellowship (2022–23, T.A. and C.A.) and a salary award (Chercheur-boursier clinicien Senior) from the Fonds de Recherche du Québec – Santé 2021 – 2023 (B.F.).

**Conflicts of interest:** None of the authors has any conﬂict of interest to disclose. Outside of this work, BF received speaker / advisory board honoraria from Eisai, Paladin labs, UCB, and UNEEG.

**Ethical Compliance:** All study participants provided written informed consent in agreement with the Research Ethics Board at the Montreal Neurological Institute (REB00010120). We confirm that we have read the Journal’s position on issues involved in ethical publication and affirm that this report is consistent with those guidelines.

Word counts:

1. Title: 99/100
2. Running head: 48/50
3. Body:2292
4. Tables:1

**Keywords:** Epilepsy, Sleep, Electrical source imaging, Interictal epileptic discharge

**Methods**

**Anatomical MRI acquisition and head model**

All patients underwent MRI scanning on a 3 Tesla Siemens Magnetom Prisma-Fit equipped with a 64-channels head coil. The acquisition included high-resolution T1-weighted magnetic resonance imaging (MRI) using a 3D magnetization - prepared rapid gradient-echo sequence (MPRAGE; 0.8 mm isovoxels, TR = 2300 ms, TE = 3.14 ms, TI = 900 ms, flip angle = 9°, FOV = 256x256 mm2). The 3T anatomical MRI was segmented, and the cortical surface was obtained using the FreeSurfer software (http://surfer.nmr.mgh.harvard.edu). The forward problem was solved using a boundary element method 1 with 3-layer for brain, skull and scalp 2 (conductivity of 0.33, 0.0165, 0.33 S/m) using OpenMEEG 3 implemented in Brainstorm. The mid surface defined as the middle layer between gray matter/pial and gray/white matter interfaces 4, together with a surface segmentation of both hippocampi, were considered as our source space for electrical source imaging (ESI).

**Electrical source imaging**

For the purpose of this study, we implemented a new depth-weighted extension of the cMEM framework ^5^, in order to better address deep seated epileptic sources. In this appendix, we will first review the main methodological concepts of the MEM framework before introducing the depth weighting implementation that was carefully evaluated by Cai et al.^6^ in functional Near Infra-Red Spectroscopy 3D reconstruction and adapted here for ESI.

For this study, we considered a depth-weighted version of cMEM ^7^, this version being able to localize more accurately deeper sources. This new improvement is paramount for this paper, as a large portion of our cohort was composed of mesiotemporal epilepsy cases (n=9). To do so, we applied the depth weighted extension of cMEM that we first proposed in Cai et al. ^6^ and we also added surfaces of bilateral hippocampi in the source space model. The activity estimates of each vertex calculated by electrical source imaging (ESI) are theoretically subject to uncertainty, which is modelled by the variance parameter in the source covariance matrix of the inverse problem. Deeper sources tend to have greater uncertainty in ESI, resulting in higher variance values in the covariance matrix when compared to superficial sources. Therefore, an a-priori source covariance matrix should appropriately account for the variance differences across vertices. To do so, the diagonal of the source covariance was weighted by the forward model of each particular vertex, quantifying the influence of source depth. The weighting was done a-priori by setting the weighting hyperparameter $\omega$ =½ (see section on depth weighting below) as a midway compromise (0 would represent no depth weighting, whereas 1 would mean prioritizing deep sources) ^8^. The noise covariance matrix was estimated using a clean 2-second segment from the same recording, this covariance was shared by all ESIs for a given patient for all vigilance states.

*Distributed sources model*

The relationship between source amplitudes and EEG measurements is expressed by the following linear model:

$\boldsymbol{M(}t\boldsymbol{)}=\boldsymbol{G}\mathbf{j}\boldsymbol{(}t\boldsymbol{)}+\boldsymbol{e(}t\boldsymbol{)}$ (1)

$\boldsymbol{M(}t\boldsymbol{)}$ is the $q$-dimensional measurement vector for EEG signal at time $t$ where $q$ denotes the number of EEG sensors, $\mathbf{j}\boldsymbol{(}t\boldsymbol{)}$ is the $r$-dimensional vector denoting current density of $r$ dipolar sources at time t and **G** is the lead field matrix with a dimension of $q\times r$. **e**(t) models an additive measurement noise at time t. We assume an anatomical constraint that the dipoles are orientated orthogonally to the surface of the cortex and hippocampus.

*MEM framework*

Within the MEM framework, the amplitude of current density $\boldsymbol{j}$ estimated for **J**, i.e. amplitude of **J** at each location in the source space (i.e., cortical and subcortical surfaces) at each time sample, is considered as a random variable, described by the following probability distribution$dp\left( \mathbf{j} \right)=p\left( \mathbf{j} \right)d\mathbf{j}$. The Kullback-Leibler divergence or $\nu$-entropy of $dp(\mathbf{j})$ relative to a prior distribution $d\nu\left( \mathbf{j} \right)$ is estimated as follows (Eq.2):

(2)

$S_{v}\left( dp \right)=-\int_{\mathbf{j}} \log\left( \frac{dp\left( \mathbf{j} \right)}{d\nu\left( \mathbf{j} \right)} \right)dp\left( \mathbf{j} \right)=-\int_{\mathbf{j}} f\left( \mathbf{j} \right) log(f\left( \mathbf{j} \right)) dv(\mathbf{j})$

where $f\left( \mathbf{j} \right)$ is the $\nu$-density of $dp\left( \mathbf{j} \right)$ defined as $dp\left( \mathbf{j} \right)=f\left( \mathbf{j} \right)d\nu\left( \mathbf{j} \right)$. Following a Bayesian approach to introduce the data fit, we denote $C_{m}$ as the set of probability distributions on j that explain the data on average (Eq. 3):

(3)

$\boldsymbol{M}-\left[ \mathbf{G} | \mathbf{I}_{q} \right]\left[ \begin{aligned} E_{dp}\left[ \mathbf{j} \right] \\ \boldsymbol{e} \end{aligned} \right] =0 ,\quad\quad dp\in C_{m}$

where $E_{dp}\left[ \mathbf{j} \right]$represents the mathematical expectation of **j** under the probability distribution $dp$, and $\mathbf{I}_{q}$ is an identity matrix of $\left( q\times q \right)$ dimension (Eq. 4) where q is the number of EEG channels . Therefore, within the MEM framework, a unique solution of $dp\left( \boldsymbol{j} \right)$ could be obtained,

(4)

$d\hat{p}^{*}=\underset{dp\in C_{m}}{\mathrm{argmax}} \left( S_{v}\left( dp \right) \right)$

The MEM solution (Eq. 5) would find a distribution of sources that maximizes the negative entropy and, thus, maximizes the missing information as previously described^9^. More specifically, to perform ESI within the MEM framework, we introduced the following prior distribution model $d\nu\left( \mathbf{j} \right)$, assuming brain activity to be organized along k independent parcels^9^:

(5)

$d\nu\left( \mathbf{j} \right)=\prod_{k=1}^{K} \left[ \left( 1-\alpha_{k} \right)\delta\left( \mathbf{j}_{k} \right)+\alpha_{k}\mathcal{N}\left( \boldsymbol{\mu}_{k},\boldsymbol{\Sigma}_{k} \right) \right]d\mathbf{j},\quad\quad0<\alpha_{k}<1$

The cortical surface was first parcellated into k parcels describing a partition of the whole surface (using data-driven parcelling^10^). The notion of coherent MEM (cMEM) was proposed in Chowdhury et al^11^, suggesting a stable spatial prior (parcellation of the brain in k parcels) along time. Each cortical parcel *k* is characterized by an activation state, defined by the hidden variable $S_{k}$, describing if the parcel is active or not. Therefore, we denote $\alpha_{k}$ as the probability of $k^{th}$ parcel to be active, i.e., $Prob\left( S_{k}=1 \right).\delta_{k}$ is a Dirac function that allows to “switch off” the parcel when considered as inactive (i.e., $S_{k}=0$). $\mathcal{N}\left( \boldsymbol{\mu}_{k},\boldsymbol{\Sigma}_{k} \right)$ is a Gaussian distribution, describing the distribution of absorptions changes within the $k^{th}$ parcel, when the parcel is considered as active ($S_{k}=1$). This prior model, which is specific to our cMEM inference, offers a unique opportunity to switch off some parcels of the model, resulting in accurate spatial reconstructions of the underlying activity patterns with their spatial extent.

*Initialization of the reference distribution (prior)*

The spatial clustering of the cortical surface into $K$ non-overlapping parcel was obtained using a data driven parcellization (DDP) technique. DDP consisted in first applying a projection method, the multivariate source prelocalization (MSP) ^12^, estimating a probability like coefficient (MSP score) between 0 and 1 for each vertex of the cortical mesh, characterizing its contribution to the data. DDP is then obtained by using a region growing algorithm, along the tessellated cortical surface, starting from local MSP maxima ^13^. Once the parcellation is done, the prior distribution $d\nu\left( \mathbf{j} \right)$ is then a joint distribution expressed as the multiplication of individual distribution of each parcel in Eq.6 assuming statistical independence between parcels, we initialize each parcel with a Gaussian distribution of the active state to be a zero mean.

To initialize the prior in Eq.5, the mean of the Gaussian distribution $\left( \boldsymbol{\mu}_{k},\boldsymbol{\Sigma}_{k} \right),$ $\boldsymbol{\mu}_{k}$, was set to zero. $\boldsymbol{\Sigma}_{k}$ at each time point, i.e. $\boldsymbol{\Sigma}_{k}\left( t \right)$ was defined as follows:

(6)

$\boldsymbol{\Sigma}_{k}\left( t \right)=\eta\left( t \right)W_{k}\left( \sigma\right)^{T}W_{k}\left( \sigma\right) ; \eta\left( t \right)=0.05\frac{1}{P_{k}}\sum_{i\in P_{k}} {\hat{\mathbf{J}}}_{MNE}{(i,t)}^{2}$

Where $W_{k}\left( \sigma\right)$ is a spatial smoothness matrix, defined by Friston et al. ^14^, which controls the local spatial smoothness within the parcel k, according to the geodesic surface. $\eta\left( t \right)$ was defined as $5\%$ of the averaged energy of Minimum Norm Estimate (MNE) solution ${\hat{\mathbf{J}}}_{MNE}$within each parcel k at time t. ($P_{k}$ being the number of vertices of the k^th^ parcel)

**Depth weighting**

*Depth weighted MNE*

Minimum Norm Estimate (MNE) ^15^ is a standard source imaging technique assessing the distribution of sources by minimizing the L2-norm of the sources amplitudes. To achieve depth weighting for MNE (dMNE) we need to initialize the source covariance matrix $\boldsymbol{\Sigma}$ as follows (Eq. 7)

(7)

${\hat{\mathbf{J}}}_{dMNE}=\arg\min\left( \left| \left| \left( \boldsymbol{M}-\mathbf{G}\boldsymbol{J} \right) \right| \right|_{\boldsymbol{\Sigma}_{d}}^{2}+\boldsymbol{\lambda}\left| \left| \boldsymbol{J} \right| \right|_{\boldsymbol{\Sigma}}^{2} \right) =\left( \mathbf{G}^{T}\boldsymbol{\Sigma}_{d}\mathbf{G}+\boldsymbol{\lambda}\boldsymbol{\Sigma} \right)^{-1}\mathbf{G}^{T}\boldsymbol{\Sigma}_{d}\boldsymbol{M}$

Where $\Sigma= diag\left( ({\mathbf{G}^{T}\mathbf{I}_{r}\mathbf{G)}}^{-\omega_{1}} \right)$

and $\mathbf{I}_{r}$is the identity matrix of dimension r (i.e. number of sources).

Depth weighted MNE (dMNE) ^8^ uses the forward model G for each vertex in the source model to weight the covariance matrix. Therefore, it appropriately represents the distribution of ESI uncertainty of each vertex across different brain regions. $\omega_{1}$ is a weighting strength parameter adjusting the amount of depth compensation. The larger is $\omega_{1}$, the more depth compensation is considered. $\omega_{1}=0$ would therefore refer to no depth compensation and an identity source covariance model.

*cMEM*

We then implemented depth weighting into the cMEM framework, following the strategy we proposed in Cai et al. ^6^ for functional Near InfraRed Spectroscopy 3D reconstruction, where we carefully evaluated the performance of the method using realistic simulations. Depth-weighted in cMEM was applied at two levels, respectively characterized by two depth weighting parameters,$\omega_{1}$ was applied to solve the depth weighted MNE, as described in Eq.6, before using those prior to initialize the source covariance model within each parcel of the cMEM model. Therefore, we used the depth weighted version of MNE solution described by Eq. 6, avoiding biasing the initialization of the source covariance with a standard MNE solution. Then $\omega_{2}$ was used to apply depth weighting on the source covariance matrix $\Sigma_{k}$ of each parcel $k$ in Eq.8. Consequently, the depth weighted version of $\Sigma_{k}\left( t \right)$ is now defined as follows:

(8)

$\boldsymbol{\Sigma}_{k}\left( t \right)_{dw}=\boldsymbol{\Lambda}_{P_{k}}\eta\left( t \right)_{dw}W_{k}\left( \sigma\right)^{T}W_{k}\left( \sigma\right); \eta\left( t \right)_{dw}=0.05\frac{1}{P_{k}}\sum_{i\in P_{k}} {\hat{\mathbf{J}}}_{dMNE}{(i,t)}^{2}$

where for each parcel k, $\Lambda_{k}=diag\left( \left( {\mathbf{G}_{k}}^{T}\mathbf{G}_{k} \right)^{-\omega_{2}} \right)$ is the depth weighting matrix for each parcel k, where $\mathbf{G}_{k}$ denotes the forward model restricted to the $P_{k}$ vertices of the kth parcel, and${\hat{\mathbf{J}}}_{dMNE}$ is the solution of depth weighted MNE (equation 6) for which we averaged the energy within the k^th^ parcel. In present implementation of depth weighted cMEM, we considered $\omega_{1}= \omega_{2}=0.5.$

**Metrics**

*Sensor level evaluation metrics*

The sensor space data were assessed using the following metrics applied on the averaged IEDs signals:

1. The absolute amplitude maximum in µVolt of the most negative channel at IED negative peak of the averaged IED.

2. IED duration from take-off to offset in ms, marked visually on the averaged IED on all channels.

3. Signal-to-noise (SNR) was assessed on the channel which displayed the highest negativity at the peak of the average IED, and defined as the mean absolute amplitude ±100ms around the IED peak divided by the standard deviation of a 200ms baseline window selected 5 seconds before the IED peak (Eq. 9)

(9)

SNR= $\frac{\frac{1}{N}\sum_{j}^{N} \left| x_{j} \right|}{\sqrt{\frac{\sum_{i}^{M} {{(y}_{i}-\frac{1}{M}\sum_{i}^{M} \left| y_{i} \right|)}^{2}}{M-1}}}$

Where, for the EEG electrode exhibiting the highest negativity, $x_{j}$ denotes the IED signal at time sample j that was averaged within a ±100ms window around the peak (N = 200 samples) and $y_{i}$ denotes for the same channel at time sample i, the baseline signal within a 200ms window (M = 200 samples).

4. Channel involvement was defined as the count of channels which exceeded a negative threshold at the time of the IED peak, which was determined by choosing the amplitude of the most negative channel at the time of the peak. The relative channel involvement was defined by using the relative threshold set as 50% of the averaged IED peak. The absolute threshold was defined by using the threshold set to 50% of the averaged IED peak considering wakefulness data as our reference for such comparison.

*Source level evaluation metrics*

After localizing the averaged IEDs in each vigilance state, ESI maps were assessed at the midpoint of the rising phase of the IED, using the following metrics:

1. Dmin: the minimum distance localization error was computed as the Euclidean distance from the maximum of the map to the closest vertex belonging to the clinical ROI (i.e. ground truth). Whenever this maximum was located inside the clinical ROI, Dmin was set to 0 mm.

2. Spatial dispersion: this metric measures the spatial spread (in mm) of the localization around the ground truth considered here as the clinical ROI. It was computed as the root mean square of the distance from the ROI weighted by the energy of the source localization map on each vertex (Eq. 1).

(10)

$Spatial dispersion=\sqrt{\frac{\sum_{i=1}^{p} ({min}_{l\in\Theta} (D_{il}^{2})J_{i}^{2}}{\sum_{i=1}^{p} J_{i}^{2}}}$

Where Θ denotes the set of vertices belonging to the clinical ROI, and $J_{i}$ is the amplitude results of the cMEM solver for the dipolar source on vertex $i$ at the midpoint of the rising phase of the averaged IED. ${Min}_{l\in\Theta} (D_{il}^{2})$ function provides the minimum Euclidean distance between the dipolar source $i$ to the closest vertex within the clinical ROI Θ. SD results are provided in mm.

3. D-maxima: when comparing ESI obtained for different states of vigilance, D-maxima was measured as the Euclidean distance between the vertices exhibiting the maximum energy in both ESI maps to be compared.

4. Spatial extent of the underlying source: the source spatial extent was assessed as the percentage of vertices identified as activated above a specific threshold. Following our previous study showing that cMEM spatial extent results are stable over a large range of thresholds^16^, benefitting from the excellent contrast of cMEM maps, we decided to apply a threshold set at 20% from maximum absolute intensity of the ESI map. We are proposing two spatial extent metrics, one relative map by setting the threshold at 20% of the source map maximum itself, and one absolute threshold set as 20% of the wakefulness source map maximum.

5. Clustering**:** To further assess the effect of the vigilance state on relative versus absolute effect of spatial extent, we examined the contribution of the amplitude variations to the ESI maps. We proposed to apply ESI using cMEM on every individual IED available, followed by a hierarchical clustering of all individual ESI maps, in a similar manner to the methodology proposed by Chowdhury et al. ^17^. To do so, we computed ESI using cMEM for each IED for all available IEDs in each vigilance state. For every IED, we only considered ESI results at one time sample, the exact peak of every IED. Every ESI map, a column vector $J^{\mu}$ estimated at the exact peak of every IED, was first normalized, by subtracting its spatial mean ($mean (J^{\mu}))$and dividing by its norm$\left\| J^{\mu} \right\|$, therefore resulting in a centred and normalized value of ESI results for every IED. We then clustered all the normalized maps into 3 clusters, and compared the resulting clustering with the true classification in NREM, REM and Wake states. We considered a hierarchical clustering approach, involving a similarity matrix $S_{\mu\nu}$consisting as the dot product between each normalized ESI map $\mu$ to every other normalized ESI map $\nu$. This proposed similarity metric was chosen to remove the influence of ESI source amplitude on the clustering, and was shown to separate IEDs based on their spatial distribution in the source space only ^17^.

(11)

$\tilde{J^{\mu}}= \frac{J^{\mu}-mean (J^{\mu})}{\left\| J^{\mu} \right\|}$, $S_{\mu\nu}= {\tilde{J^{\mu}}}^{T}.\tilde{J^{\nu}}$

Where $T$ denotes the transposition of the column vector $\tilde{J^{\mu}}$.

We then we repeated this clustering process but this time we added an additional term to the similarity matrix representing the amplitude of the ESI maps. This amplitude was the squared difference of the norms between each two maps $A_{uv}$. This additional term was normalized to have a maximum of 1 in each row ($\tilde{A}_{uv})$, such that the two most different maps in term of amplitude will have a value of one. This term was then multiplied by a regularization parameter equal to λ = 0.1 to consider a small contribution of the amplitude differences when compared to the similarity in spatial profile (Eq. 11, 12).

(12)

${A_{uv}=\left( \left\| J^{\mu} \right\|-\left\| J^{\nu} \right\| \right)^{2};\tilde{A}_{uv}=\frac{A_{uv}}{{max}_{u} (A_{uv})};and similarity metric ;S}_{\mu\nu}={\tilde{J^{\mu}}}^{T}.\tilde{J^{\nu}}+\lambda A_{uv}$

Where λ is a fixed parameter. The λ parameter was tuned on a subset of 5 patients. Using this new metric two ESI maps were considered closed together if they were exhibiting similar spatial features and similar amplitudes. This allowed us to check whether the clustering changed when an amplitude information was introduced. If the amplitude bares no effect on the ESI, then the two clustering methods should be similar.

6. Sub-lobar clinical analysis: the ESI maps were reviewed by an epileptologist and determined on a sub-lobar level if the peak of the map is concordant or discordant with the clinical ground truth.

Unequalized condition

|  | **N2** | **N3** | **REM** | **Wake** | **Clinical** |
| --- | --- | --- | --- | --- | --- |
| # interictal epileptiform discharges | 24 [6-102] | 26 [17-226] | 16 [6-55] | 14.5 [5-77] | 81 [18-223] |
| IED peak amplitude (µV) | 58.99[33.45-76.49] | 67.39 [37.58-98.78] | 27.82 [18.86-43.08] | 36.61 [21.81-82.60] | 49.26 [32.54-89.37] |
| Average IED duration (ms) | 71.56±31.8 | 83.25±30.4 | 71.8±28.42 | 62.19±21.79 | 73.0±26.02 |
| Signal-to-noise ratio | 8.56 [4.85-13.39] | 11.93 [6.52-27.84] | 5.74 [3.43-9.05] | 4.69 [3.98-7.75] | 13.46 [9.18-22.67] |
| # involved sensor (relative threshold) | 13.5 [9.75-15] | 12.5 [ 9-14.75] | 13.5 [9.5-18] | 14 [11-15.25] | 13 [11-14] |
| # involved sensor (absolute threshold) | 15.5 [11-18.75] | 16.5 [13-21.5] | 11.5 [3-18.5] | Reference | 18.5 [10.5-22.5] |
| Minimum distance (mm) | 7.18±13.27 | 16.82±11.67 | 12.69±12.61 | 16.11±13.00 | 16.32±10.68 |
| Spatial dispersion (mm) | 18.69±6.4 | 18.4±6.3 | 19.14±7.88 | 18.78± 6.72 | 19.88±6.94 |
| % of activated vertices (relative threshold) | 1.38±0.54 | 1.46±0.63 | 1.59±0.54 | 1.52±0.7 | 1.71±0.8 |
| % of activated vertices (absolute threshold) | 1.57 [0.29-2.76] | 1.76 [1.105-2.96] | 0.26 [0-1.105] | Reference | 2.38 [1.37-3.20] |
|  |  |  |  |  |  |

Equalized condition

|  | **N2** | **N3** | **REM** | **Wake** | **Clinical** |
| --- | --- | --- | --- | --- | --- |
| IED peak amplitude (µV) | 51.3 [30.97-72.09] | 55.39 [37.02-95.51] | 28.35 [21.44-41.86] | 37.56 [24.67-74.07] | 60.27 [38.37-75.01] |
| Average IED duration (ms) | 84.12±36.93 | 83±30.34 | 71.06±24.36 | 59. ±25.43 | 77.12±40.82 |
| Signal-to-noise ratio | 5.1 [ 3.73-9.69] | 5.98 [3.77-8.41] | 4.23 [3.06-6.07] | 4.46 [2.72-7.41] | 7.12 [2.97-10.09] |
| # involved sensor (relative threshold) | 13 [11.25-15] | 12.5 [9.75-14] | 14.5 [9.5-19.25] | 12.5 [8.75-14.25] | 12.5 [11-14] |
| # involved sensor (absolute threshold) | 15 [10.75-21.5] | 16.5 [11.75-18.5] | 11 [3-14.25] | Reference | 14.5 [11-14] |
| Minimum distance (mm) | 15.85±10.72 | 15.07±11.75 | 15.73±12.86 | 14.81±12.47 | 15.18±10.01 |
| Spatial dispersion (mm) | 18.93±6.0 | 18.05±5.72 | 19.98±6.66 | 17.98±6.01 | 16.78±5.7 |
| % of activated vertices (relative threshold) | 1.46±0.74 | 1.94±0.91 | 1.65±0.67 | 1.65±0.82 | 1.72±0.86 |
| % of activated vertices (absolute threshold) | 2.16 [1.22-3.86] | 3.07 [1.86-5.04] | 0.82 [0-1.9375 ] | Reference | 2.31 [0-9.15] |

**Table 1.** All the values measured for each metric.

**SI References**

1. Kybic J, Clerc M, Faugeras O, Keriven R, Papadopoulo T. Generalized head models for MEG/EEG: boundary element method beyond nested volumes. Physics in medicine and biology. 2006;51:1333-46.

2. Lai Y, van Drongelen W, Ding L, et al. Estimation of in vivo human brain-to-skull conductivity ratio from simultaneous extra- and intra-cranial electrical potential recordings. Clin Neurophysiol. 2005 Feb;116(2):456-65.

3. Gramfort A, Papadopoulo T, Olivi E, Clerc M. Forward field computation with OpenMEEG. Comput Intell Neurosci. 2011;2011:923703.

4. Fischl B, Salat DH, Busa E, et al. Whole brain segmentation: automated labeling of neuroanatomical structures in the human brain. Neuron. 2002 Jan 31;33(3):341-55.

5. Chowdhury RA, Merlet I, Birot G, et al. Complex patterns of spatially extended generators of epileptic activity: Comparison of source localization methods cMEM and 4-ExSo-MUSIC on high resolution EEG and MEG data. NeuroImage. 2016.

6. Cai Z, Machado A, Chowdhury RA, et al. Diffuse optical reconstructions of functional near infrared spectroscopy data using maximum entropy on the mean. Sci Rep. 2022 Feb 10;12(1):2316.

7. Afnan JC, Z; Lina, Jean-M; Hedrich, Tanguy; Abdallah, C; Avigdor, T; von Ellenrieder, N; Frauscher, B; , Gotman JG, C. Localizing deep generators of epileptic activity using depth weighted Maximum Entropy on the Mean. Organization for Human Brain Mapping (OHBM)2023.

8. Lin FH, Witzel T, Ahlfors SP, Stufflebeam SM, Belliveau JW, Hamalainen MS. Assessing and improving the spatial accuracy in MEG source localization by depth-weighted minimum-norm estimates. Neuroimage. 2006 May 15;31(1):160-71.

9. Amblard C, Lapalme E, Lina JM. Biomagnetic Source Detection by Maximum Entropy and Graphical Models. IEEE Transactions on Biomedical Engineering. 2004;51:427-42.

10. Chowdhury RA, Merlet I, Birot G, et al. Complex patterns of spatially extended generators of epileptic activity: Comparison of source localization methods cMEM and 4-ExSo-MUSIC on high resolution EEG and MEG data. NeuroImage: Academic Press Inc.; 2016. p. 175-95.

11. Chowdhury RA, Lina JM, Kobayashi E, Grova C. MEG Source Localization of Spatially Extended Generators of Epileptic Activity: Comparing Entropic and Hierarchical Bayesian Approaches. PLoS ONE2013.

12. Mattout J, Pelegrini-Issac M, Garnero L, Benali H. Multivariate source prelocalization (MSP): use of functionally informed basis functions for better conditioning the MEG inverse problem. Neuroimage. 2005 Jun;26(2):356-73.

13. Lapalme E, Lina JM, Mattout J. Data-driven parceling and entropic inference in MEG. Neuroimage. 2006 Mar;30(1):160-71.

14. Friston K, Harrison L, Daunizeau J, et al. Multiple sparse priors for the M/EEG inverse problem. Neuroimage. 2008 Feb 1;39(3):1104-20.

15. Hamalainen MS, Ilmoniemi RJ. Interpreting magnetic fields of the brain: minimum norm estimates. Med Biol Eng Comput. 1994 Jan;32(1):35-42.

16. Pellegrino G, Hedrich T, Porras-Bettancourt M, et al. Accuracy and spatial properties of distributed magnetic source imaging techniques in the investigation of focal epilepsy patients. Human Brain Mapping. 2020.

17. Chowdhury RA, Pellegrino G, Aydin Ü, et al. Reproducibility of EEG-MEG fusion source analysis of interictal spikes: Relevance in presurgical evaluation of epilepsy. Human Brain Mapping. 2018.
